# Editorial: Identification of potential therapeutic targets for the tumor microenvironment of gastrointestinal tumor

**DOI:** 10.3389/fimmu.2024.1442608

**Published:** 2024-06-27

**Authors:** Zhiliang Li, Yimei Jiang, Kun Liu, Ren Zhao

**Affiliations:** ^1^ Department of General Surgery, Ruijin Hospital, Shanghai Jiao Tong University School of Medicine, Shanghai, China; ^2^ Shanghai Institute of Digestive Surgery, Ruijin Hospital, Shanghai Jiao University School of Medicine, Shanghai, China

**Keywords:** tumor microenvironment, tumor therapy, gastrointestinal tumors, therapeutic target identification, tumor treatment prediction

Cancer is recognized as a progressive and interactive process, involving constant, dynamic, and reciprocal interactions between cancer cells and the tumor microenvironment (TME). The TME is composed of many different cellular and acellular components that together influence tumor growth, invasion, metastasis, and its response to different therapeutic regimens, which include fibroblasts, endothelial cells, neurons, adipocytes, immune cells, lymphatics, and vasculature, in addition to its non-cellular components like the extracellular matrix (ECM) and secreted products such as chemokines, cytokines, growth factors, and extracellular vesicles (1–4). To date, immunotherapy, ranging from immune-checkpoint blockade therapy to adoptive cell therapy and tumor vaccines, has displayed considerable success in many gastrointestinal tumors such as microsatellite instability-high (MSI-H) colorectal cancer (CRC), gastric cancer, and hepatocellular carcinoma (5–8). However, only a small fraction of patients could benefit from immunotherapy. Resistance to standard adjuvant chemotherapy and radiotherapy posed urgent scientific problems to be solved (9, 10). In order to improve the anti-cancer therapeutic benefit, many efforts have been devoted to exploring the novel targets of anti-cancer therapy in the TME and achieving its clinical translation. This topic collected six scientific studies focused on the identification of potential therapeutic targets for the TME of gastrointestinal tumors.

In the interaction between tumor cells and TME, macrophages under hypoxic conditions are acknowledged as a pivotal determinant in the progression of CRC. Jiang et al. discovered that hypoxia-induced macrophage-derived exosomes (HMDEs) can induce cell cycle transition and inhibit cell apoptosis, thereby promoting the growth of CRC cells. Additional molecular mechanisms illustrate that the overexpression of Hif-1α interacts with specific regions (-521− -516 bp and -401− -391 bp) of the Hsp90 promoter under hypoxic conditions, resulting in an increase in Hsp90 protein levels within HMDEs. The interaction between upregulated Hsp90 and the large tumor suppressor kinase 1 (Lats1) facilitates the deactivation of Lats1 and the inhibition of Yap phosphorylation, leading to the ultimate inactivation of the Hippo signaling pathway. By inhibiting the Hippo signaling pathway, these findings unveil the crucial role of HMDEs Hsp90 in promoting CRC progression, which indicates that Hsp90 may be a potential therapeutic target for CRC. In the clinical treatment of CRC, distant metastasis is an intractable and significant field in addition to tumor growth and local invasion. Wu et al. focused on the underlying relationship between gut microbiota and CRC metastasis. The authors suggest that liver metastasis to primary CRC is possibly due to the formation of a pre-metastatic niche (PMN) in the liver, which is characterized by the infiltration of immunosuppressive cells and increased pro-inflammatory immune responses. They highlight the multiple functions of different immune cell types in the TME, especially neutrophils and neutrophil extracellular traps (NETs) in the TME. This review summarizes the possible mechanisms of gut microbiota in shaping PMN formation, providing therapeutic indications for the TME in clinical CRC treatment. Meanwhile, Ding et al. provided an overview of the current research progress on the TME characteristics in terms of immune cells, cancer-associated fibroblasts (CAFs) and the gut microbiota in the TME respectively. Moreover, the authors also summarized the advancements in immunotherapy for MSS CRC, especially the different therapeutic strategies and biomarkers of ICIs, Chimeric antigen receptor T cell (CAR-T) therapy and cancer vaccines in MSS CRC, offering us new insights to improve the efficacy and identify the potential targets of immunotherapy.

To evaluate the role that cytokines may play in GC in relation to angiogenesis, metastasis, and chemoresistance, Reyes et al. drew conclusions on the relationship between cytokine-associated epigenetic regulation and its potential effects for the different stages of development and chemoresistance in GC. Epigenetic alterations and silencing (e.g. DNA hypermethylation, histone deacetylase) affect key genes and pathways (e.g. expression of CXCL12 and its receptor) associated with immune response, tumor angiogenesis and cytokine secretion, all of which are essential for tumor growth and metastasis in GC progression. A comprehensive understanding of the epigenetic and cytokine landscape of the TME in GC encourages us to believe that the use of DNA methyltransferase and histone deacetylase inhibitors is a promising therapeutic approach in GC. Meanwhile, in order to analyze the heterogeneity of the TME, especially the tumor immune microenvironment (TIME), Mou et al. provided a more thorough understanding of the biological mechanisms behind tumor-associated macrophages, tumor-associated neutrophils, and natural killer cells at the single-cell level. This review article offers us fresh, innovative insights on identifying new targets to increase the clinical efficacy of GC therapy.

Pancreatic ductal adenocarcinoma (PDAC) is one of the most lethal cancers in gastrointestinal tumors. However, the therapeutic challenges lie in the late diagnosis and establishment of the peculiar TME, which is heterogeneous between patients and even within the same patient. By organizing functional sub-TME and highlighting recent advances, Musiu et al. conclusively deciphered the TME landscape and CAF heterogeneity in PDAC, both of which play a pivotal role in supporting or dampening pancreatic cancer progression. Taking advantage of spatial biology (novel multi-omics plus imaging technologies), the authors reviewed cellular interactions within the tissue architecture of the TME in PDAC. Finally, the researchers provided context and illustrated how clinics will translate the acquired knowledge to design new-generation clinical trials that promise to improve the efficacy of conventional and immune-based therapies.

Focusing on the molecular mechanisms and cellular interactions within the TME, these six scientific studies are devoted to identifying the potential therapeutic targets for gastrointestinal tumors with the ultimate goal of improving the clinical outcomes of patients. We would like to express our sincere gratitude to all the authors, reviewers, editors, topic editors and editorial team of *Frontiers in Immunology* for their dedication and assistance in the process of reviewing and publishing all these studies in this Research Topic. We believe that with the explorations and efforts dedicated to TME, anti-cancer targeted therapies and comprehensive treatment management for gastrointestinal tumors will enter a whole new era in the near future.

Targets identification, Tumor Treatment Prediction

## Author contributions

ZL: Writing – original draft, Writing – review & editing. YJ: Writing – original draft, Writing – review & editing. KL: Writing – original draft, Writing – review & editing. RZ: Writing – original draft, Writing – review & editing.
